# Persistence of Symptoms and Long-Term Recovery in Hospitalized COVID-19 Patients: Results from a Five-Year Follow-Up Cohort

**DOI:** 10.3390/idr18010008

**Published:** 2026-01-09

**Authors:** Ana Roel Conde, Francisco Javier Membrillo de Novales, María Navarro Téllez, Carlos Gutiérrez Ortega, Miriam Estébanez Muñoz

**Affiliations:** 1 University of Alcalá, Plaza de San Diego, 28801 Alcalá de Henares, Madrid, Spain; 2 Department of Internal Medicine, Hospital Central de la Defensa “Gómez Ulla”, 28047 Madrid, Madrid, Spain; 3 CBRN and Infectious Diseases Department, Hospital Central de la Defensa “Gómez Ulla”, 28047 Madrid, Madrid, Spain

**Keywords:** SARS-CoV-2 pneumonia, post-COVID-19 condition, PCC, risk factors

## Abstract

Background/Objectives: This study aimed to determine the prevalence of persistent symptoms and the radiological and laboratory evolution at 6 months and 5 years after discharge in patients hospitalized for SARS-CoV-2 pneumonia during the first wave of the pandemic in Spain and to estimate the healthcare impact of their follow-up. Methods: A retrospective longitudinal observational study was conducted at the “Hospital Central de la Defensa”. A total of 200 patients aged >18 years with a diagnosis of SARS-CoV-2 pneumonia were screened. Clinical, radiological, and laboratory data were collected from electronic medical records. Patients with symptoms or radiological abnormalities at discharge underwent in-person evaluations, while the remainder were assessed by telephone. Results: A total of 182 patients met the inclusion and exclusion criteria. Of these, 112 were assessed in the outpatient setting; 60.7% required in-person evaluations, with normal pulmonary auscultation in 93.6%, complete radiological resolution in 85%, and normalized laboratory parameters in almost all cases. At 6 months, 26.5% presented at least one residual symptom, whereas only three patients (4.5%) reported symptoms at 5 years. No risk factors associated with symptom persistence were identified. The estimated cumulative healthcare cost was EUR 21,627.50. Conclusions: Among patients hospitalized for SARS-CoV-2 pneumonia during the first wave of the pandemic, 26.7% and 4.46% presented at least one persistent symptom at 6 months and 5 years after discharge, respectively.

## 1. Introduction

The term “post-COVID-19 condition” (PCC), also commonly referred to as long COVID, refers to a range of signs and symptoms that appear or persist after the acute phase of SARS-CoV-2 infection, most commonly including fatigue, often exacerbated by physical or mental exertion, dyspnea, muscle and joint pain, chronic cough, and cognitive disturbances such as brain fog, impaired concentration, and memory problems [1,2,3,4]. According to the World Health Organization (WHO), PCC is characterized by symptoms that usually start within three months of the initial COVID-19 illness, last for at least two months, and cannot be explained by an alternative diagnosis [5]. It is a multisystem condition with a highly variable presentation. Currently, there is no unified consensus among international institutions regarding its definition, particularly concerning the terminology and the temporal criteria considered [6]. This lack of homogeneity, together with the nonspecific nature of many symptoms, hampers comparison across studies and the drawing of solid conclusions.

The World Health Organization (WHO) estimates that the prevalence of PCC in the general population ranges between 10% and 20%. However, these figures have been progressively revised, especially following the introduction of vaccination, with reported rates decreasing from 50 to 70% in hospitalized patients during the first pandemic waves to around 10% in the post-vaccination period [7]. It is estimated that 400 million people worldwide are affected [8].

The etiopathogenic mechanisms and precise risk factors of PCC have not been conclusively characterized, with considerable discrepancies in the literature that limit the development of optimal and specific therapeutic approaches [9]. To date, the severity of the acute COVID-19 episode remains one of the most consistently supported risk factors [10,11,12] along with other factors such as sex, age, and the presence of comorbidities [13,14,15]. This is closely related to both vaccination status and viral variant, which have also been identified as key determinants influencing disease severity and long-term outcomes [9,16]. Patients affected during the first waves, before the vaccination campaign and lack of immunity by natural infection, tended to experience more severe acute disease, which may partly explain the higher incidence of PCC observed in this population [17,18]. In particular, patients infected with the wild-type strain were managed according to non-standardized treatment protocols, potentially leading to unknown long-term consequences [19]. In addition, available evidence supports that the early SARS-CoV-2 variants are, per se, associated with a higher risk of developing post-COVID-19 condition compared with the Omicron variant, independently of vaccination status [20]. Nevertheless, other authors point out that, despite this lower individual risk, the very high number of Omicron infections has resulted in a substantial absolute burden of patients with persistent symptoms [21].

Further scientific evidence is needed to better characterize this condition and to guide healthcare resource allocation and follow-up strategies, considering its significant socioeconomic impact and the large number of individuals still affected worldwide.

In this context, the present study analyzes a cohort of patients hospitalized for COVID-19 pneumonia in a tertiary hospital, defined as a referral center providing specialized and advanced medical care, during the first pandemic wave in Spain, with follow-up assessments at 6 months and 5 years after discharge. The main objective was to determine the prevalence and nature of persistent symptoms at both time points. Secondary objectives included evaluating radiological and laboratory outcomes, identifying potential risk factors for PCC, and estimating the cumulative healthcare impact.

## 2. Materials and Methods

A longitudinal, retrospective, observational study was conducted. All patients hospitalized between February and March 2020 at the Central Defense Hospital “Gómez Ulla” were identified and reviewed through the electronic medical record system. A consecutive, non-probabilistic sampling method was applied.

Patients aged over 18 years who had a discharge diagnosis of SARS-CoV-2 pneumonia confirmed by a positive PCR test, or with compatible clinical and radiological findings, were included, in accordance with national recommendations at that time [22]. Patients whose medical records revealed alternative diagnoses or those with nosocomial SARS-CoV-2 pneumonia were excluded.

Clinical data from the hospitalization period and from a specific post-COVID-19 follow-up clinic created for these patients at the Central Defense Hospital were collected. The first assessment was conducted by telephone, during which the need for an in-person evaluation was determined using a structured checklist (Supplementary File S1). Patients who reported persistent symptoms during the telephone assessment or who presented radiological abnormalities on their last chest X-ray prior to discharge were reevaluated on an outpatient basis with clinical, laboratory, and radiological follow-up, according to a standardized protocol. Only symptoms that the attending physician attributed to PCC and recorded in the electronic medical record were considered. Patients could continue follow-up in this clinic or be referred to other specialists if necessary. To evaluate the symptoms attributed to long COVID during the long-term follow-up, including the 5-year assessment, the symptoms documented by specialists in the specific post-COVID-19 clinic at the Central Defense Hospital were considered. These diagnoses were included in the medical reports as part of the clinical assessment and were verified to meet the WHO criteria for PCC, which requires that symptoms appear within three months of the acute infection, persist for at least two months, and cannot be explained by alternative diagnoses, as mentioned in the Introduction.

The economic analysis was designed as an exploratory subanalysis aimed at estimating the cumulative healthcare impact of patient follow-up. To this end, the number of medical visits and complementary tests performed was recorded, and official tariffs published in the Boletín Oficial del Estado (Official State Gazette, BOE No. 105, 1 May 2025) were applied.

The study was approved by the Ethics Committee of the Central Defense Hospital “Gómez Ulla”, which granted a waiver of informed consent due to the retrospective nature of the study and the restrictive circumstances of the pandemic. Data were obtained from the electronic medical record system (HCIS), and when information was unavailable, the HORUS platform was consulted to access records from primary care or other healthcare centers.

Statistical analysis was performed using SPSS^®^ software version 25 (IBM, Armonk, NY, USA). Both descriptive and analytical procedures were applied according to the nature of the variables. For quantitative variables, depending on whether the assumption of normality was met (assessed using the Kolmogorov–Smirnov test), data were expressed as mean and standard deviation (SD) for normally distributed variables or as median (Md) and interquartile range (IQR) otherwise. Categorical variables were summarized as absolute and relative frequencies (percentages).

To explore potential risk factors associated with the development of PCC, the chi-square test was used for categorical variables, and Student’s *t*-test or the Mann–Whitney U test was applied for quantitative variables, as appropriate. Chi-square tests were applied exclusively for exploratory purposes. No multivariate or adjusted analyses were performed due to sample size constraints. Odds ratios and 95% confidence intervals were not reported because the small cell sizes in the contingency tables would not yield reliable estimates.

Generative artificial intelligence (ChatGPT, GPT-5.2, OpenAI, San Francisco, CA, USA) was used solely to assist with minor grammatical and stylistic improvements. No AI tools were used for data analysis, interpretation, or content generation.

## 3. Results

**Figure 1:**
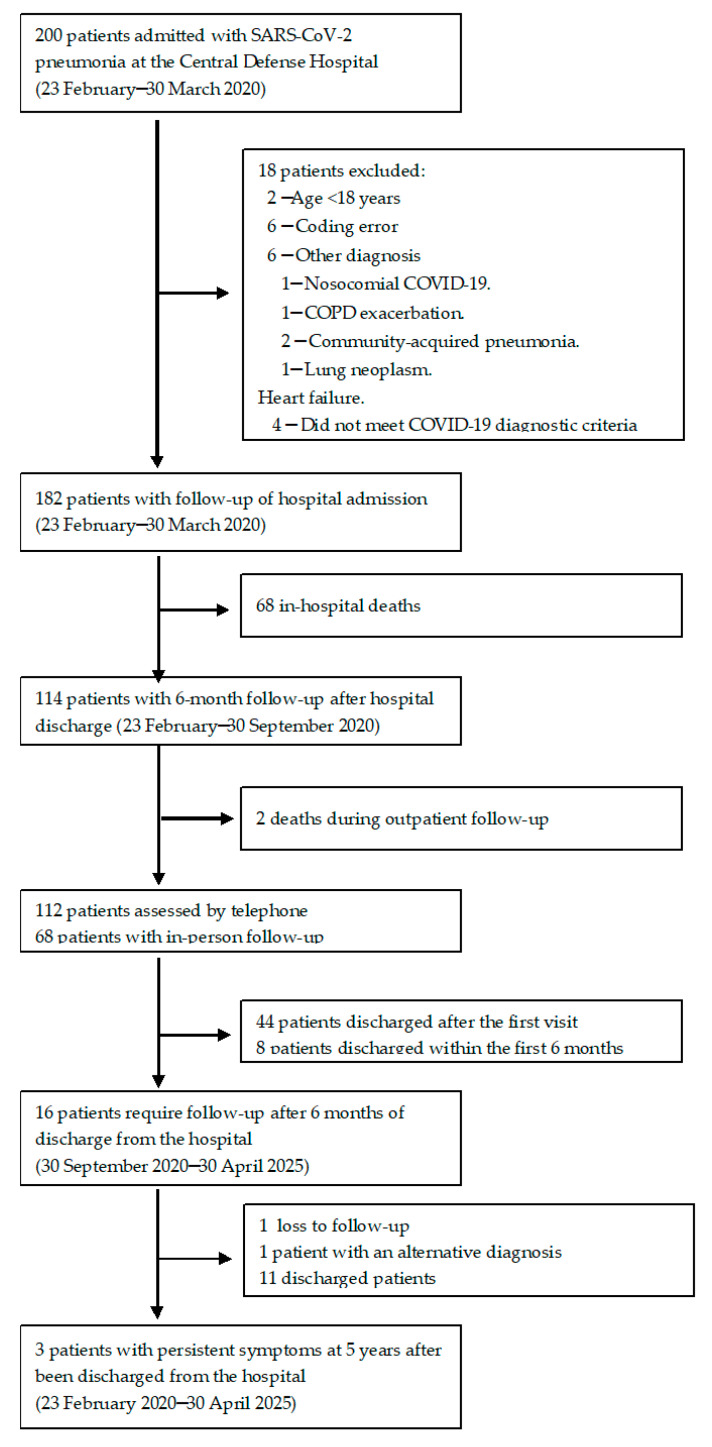
Flow diagram illustrating the inclusion, exclusion, and follow-up process of patients hospitalized with SARS-CoV-2 pneumonia during the first pandemic wave.

During the study period, 200 patients were screened. After reviewing medical records, 182 patients with a diagnosis of SARS-CoV-2 pneumonia met the inclusion criteria, and none met the exclusion criteria. Among them, 68 patients (37.3%) died during hospitalization. Two additional patients (1.6%) died within the six months following hospital discharge (Figure 1).

### 3.1. Clinical Assessment of Patients

A total of 112 patients were evaluated in the post-COVID-19 follow-up clinic by telephone. Among them, 60.7% (*n* = 68) required in-person follow-up at a mean of 119 days (*SD* = 101.3) after hospital discharge. Table 1 summarizes the clinical, radiological, and laboratory variables recorded during the first in-person assessment.

Overall, 26.5% *(n* = 30) of patients reported at least one residual symptom. Respiratory complaints were the most frequent, followed by myalgia and headache. On physical examination, pulmonary auscultation revealed preserved vesicular breath sounds in 93.6% of patients, while 6.4% presented with crackles. The mean baseline oxygen saturation was 97.5% (*SD* = 1.48).

Regarding radiological follow-up, 85% of patients showed complete resolution of lung lesions, whereas 14.5% exhibited improvement of infiltrates without deterioration. In the follow-up laboratory tests, mean inflammatory and prothrombotic markers were within normal ranges at six months; therefore, no further routine follow-up was deemed necessary.

Table 2 compares patients with persistent symptoms (n = 30) and asymptomatic patients (*n* = 82). No statistically significant differences were found between the two groups in anthropometric variables, pre-existing comorbidities, specific treatments received for SARS-CoV-2 pneumonia, length of hospital stay, proportion of patients requiring supplemental oxygen, or mean values of inflammatory and coagulation markers obtained during hospitalization.

Among the 68 patients with in-person follow-up, 64.7% (*n* = 44) were discharged after the first visit, and an additional 11.8% (*n* = 8) within the first six months. The remaining 23.5% *(n* = 16) required prolonged follow-up, with a median of 144 additional days (IQR = 1016) beyond the first six months and a mean of 2.16 (*SD* = 2.56) extra visits in the PCC clinic (range: 1–12).

Follow-up was completed once patients achieved resolution of symptoms and normalization of complementary tests. Only one patient was lost to follow-up, and one was discharged due to an alternative diagnosis of lung cancer and subsequently referred to oncology.

At five years, only three patients (4.46%) continued to experience symptoms, two of whom remained under active follow-up. Table 3 summarizes their baseline characteristics, the acute episode of SARS-CoV-2 pneumonia, findings from the initial post-COVID-19 assessment, and the long-term follow-up data.

One patient presented with persistent dyspnea and cough since the acute phase and developed arthralgia during the fourth year of follow-up. Another maintained constant anosmia and ageusia from onset, accompanied by fluctuating fatigue; this patient was eventually discharged during follow-up. The third patient developed post-COVID-19 myasthenia gravis and anterior ischemic optic neuropathy (AION) associated with post-COVID-19 choroiditis.

### 3.2. Healthcare Cost Assessment

During the follow-up period, a total of 177 evaluations were performed in the post-COVID-19 outpatient clinic, with a unit cost of EUR 84.98, resulting in a total expenditure of EUR 11,633.03.

Thirty-one referrals to other specialties were made, generating an accumulated cost of EUR 2634.38. Various complementary tests were requested according to patients’ symptoms and clinical evolution, with a total cost of EUR 7360.09. The complete distribution of referrals and diagnostic tests is presented in Table 4.

The overall estimated healthcare expenditure for post-COVID-19 follow-up in this cohort amounted to EUR 21,627.50, corresponding to an average cost of approximately EUR 318 per patient who required in-person follow-up.

## 4. Discussion

SARS-CoV-2 infection has generated great interest regarding its medium- and long-term sequelae. The prevalence and characteristics of post-COVID-19 symptoms vary considerably across studies, particularly during the first pandemic waves and the pre-vaccination era, as in the case of our cohort. Follow-up in post-COVID-19 clinics has emerged as a key tool to identify, evaluate, and manage these sequelae.

In the present cohort, 26.7% of patients evaluated in the post-COVID-19 clinic reported at least one symptom six months after discharge, a proportion lower than that described by other authors in hospitalized patients from the same period [23]. This difference may be partly explained by the severity of the acute episode. Huang et al. published an observational study with a six-month follow-up in hospitalized COVID-19 patients [24]. In their cohort, the mean duration of hospital stay was 14 days; 68% required oxygen therapy via nasal cannula or Venturi mask, and 11% required high-flow nasal cannula, noninvasive ventilation, or invasive mechanical ventilation. In that study, 76% of patients reported at least one persistent symptom, with those who required intensive care showing a higher prevalence of long-term symptoms compared with patients who did not require respiratory support.

In our cohort, in-hospital mortality was 37%, similar to other Spanish series from the first wave, which reported rates around 28% [25]. However, our patients had shorter hospital stays and lower oxygen requirements, indicating milder clinical courses than those described in the study. These cases correspond to the earliest phase of the pandemic, when admission criteria were more lenient, just before the collapse of the healthcare system. Consequently, many of these patients might have been managed on an outpatient basis. The incidence of PCC among non-hospitalized and non-vaccinated patients during the first wave is between 10 and 37% [21,26]. Given that, globally, most COVID-19 cases were mild and managed on an outpatient basis, the absolute number of individuals with PCC is higher among those with initially mild disease compared with hospitalized patients [27,28]. For this reason, the NICE guidelines on PCC management emphasize the need to consider all patients, regardless of the severity of the acute illness [29].

Respiratory symptoms were the most frequent findings during post-COVID-19 follow-up in our cohort. The absence of anosmia and the low prevalence of “brain fog,” commonly reported by other authors, are notable [30,31]. In our study, symptoms were collected via telephone interviews with open-ended questions, followed by an in-person clinical evaluation. This method may have underestimated some symptoms, as patients might not spontaneously report them, and physicians did not use standardized questionnaires.

No specific factors were associated with the presence of persistent symptoms at six months. Several studies have identified age as a potential risk factor for developing PCC [32,33]. However, recent evidence suggests that the severity of the acute episode has a stronger influence on the development of PCC than age itself [34]. In a prospective multicenter analysis by V. Daitch et al., age was not an independent predictor of symptom persistence at five months, highlighting the influence of other variables such as in-hospital complications or pre-existing comorbidities [35]. Our findings are consistent with these results.

Regarding sex differences, our study observed a higher absolute number of symptomatic men, consistent with the distribution at admission. In contrast, Notarte et al. published a 2022 meta-analysis identifying female sex as a significant risk factor for PCC, with an odds ratio (OR) of 1.48 (95% CI: 1.17–1.86, *p* = 0.01) and moderate heterogeneity across studies [9]. This finding has been supported by multiple investigations, though methodological biases have also been noted [13,15]. Some authors suggest that women may be more likely to report symptoms or seek medical attention [36], a phenomenon documented in the literature on gender differences in healthcare access, which could bias results by increasing the apparent prevalence in women [37].

Regarding treatments used during the first wave, hydroxychloroquine and azithromycin were administered more frequently among symptomatic patients, though without statistically significant differences. The available literature on this aspect is limited. Among the pharmacological treatments evaluated, only the use of remdesivir has been associated with a significant reduction in persistent symptoms in some studies [19,38,39].

Complementary tests revealed complete or partial radiological resolution of lung lesions and normalization of laboratory parameters in most patients. Berry C. et al. evaluated 443 patients in nine months and compared them with controls from the general population [40]. They found no significant radiological abnormalities among patients with mild COVID-19 and reported similar NYHA functional class between groups. Currently, no reliable biomarkers have been identified for the detection of PCC, either during hospitalization or in follow-up [41]. Some authors have suggested that D-dimer could aid in post-discharge monitoring, although the evidence remains limited [42]. It is important to note that the absence of laboratory or imaging abnormalities does not exclude the presence of persistent clinical symptoms, as documented in numerous studies [43].

To date, no studies have reported five-year follow-up outcomes. Vallée et al. published a prospective cohort study with a 3.5-year follow-up of 85 patients, in which 25% continued to experience symptoms consistent with PCC [44]. Although symptom severity improved over time, patients reported a lower quality of life than controls.

In our cohort, only three patients continued to experience symptoms attributable to PCC five years after discharge, according to the assessment of the responsible physicians. Their profiles were highly heterogeneous in terms of age, all with a history of mild pneumonia and no relevant comorbidities. Two patients had persistent abnormal lung auscultation findings, and one had residual radiological abnormalities, with no laboratory alterations during the first post-COVID-19 assessment. These data highlight that the assessment of persistent symptoms, ranging from psychological to somatic manifestations, is complex in clinical practice and that patients should be managed in specialized post-COVID-19 clinics.

Recent guidelines, such as those issued by the American Academy of Physical Medicine and Rehabilitation (AAPM&R) [45], recommend a holistic evaluation starting three months after the acute phase. They emphasize the importance of validating patients’ symptoms, performing targeted physical examinations, and maintaining a proactive diagnostic approach to identify alternative or concomitant pathologies.

These findings suggest a general trend toward spontaneous recovery while underscoring the clinical heterogeneity of the syndrome and the challenge of predicting its long-term course.

In this study, the mean time to spontaneous clinical resolution was 11 months after hospitalization, consistent with a meta-analysis including 1.2 million cases, which reported a mean of nine months among hospitalized patients (95% CI: 7–12). In that analysis, 15.1% of patients remained symptomatic after 12 months [46]. In our cohort, approximately half of the patients who were symptomatic at six months required follow-up beyond the initial visit, reinforcing the importance of monitoring patients with persistent early symptoms.

Although guidelines such as NICE [29] and AAPM&R [47] provide recommendations for PCC management, no standardized follow-up model currently exists, and the wide variability of symptoms complicates the design of uniform protocols. This situation carries significant healthcare and economic implications [48].

Beyond the individual clinical burden, PCC has been associated with a substantial socioeconomic impact at a population level, affecting healthcare systems, labor participation, and productivity [49]. Recent estimates suggest that millions of individuals worldwide experience reduced work capacity or prolonged work absence due to persistent post-COVID-19 symptoms, resulting in significant indirect costs related to loss of productivity and increased reliance on social support systems [8,49]. Economic analyses have estimated that the annual global economic toll of PCC may reach approximately 1% of the global gross domestic product, highlighting its relevance as a public health and economic challenge [8].

In the United Kingdom, a matched cohort study was conducted, identifying 52,988 individuals with a diagnosis of long COVID and 264,867 matched controls without long COVID. Healthcare utilization was assessed across primary care consultations, prescriptions, hospital admissions, emergency department visits, and outpatient appointments. During a 12-month follow-up, patients with long COVID showed a 49% higher overall use of healthcare services and approximately twice as many annual visits compared with controls (around 30 versus 16 visits per person per year), with statistically significant differences. Additionally, individuals with long COVID were more likely to incur healthcare expenditures and had 44% higher costs, with an average annual cost of approximately GBP 2500 per patient, compared with GBP 1500 in the comparator group [50].

David Cutler, in 2022, estimated the overall economic burden at USD 3.7 trillion in the United States. Nearly 60% of this cost was attributed to loss of quality of life, while the remaining proportion was related to decreased earnings and increased healthcare expenditure [49].

In our study, the total estimated healthcare expenditure exceeded EUR 21,000, with an average cost of EUR 318 per patient requiring in-person follow-up. Of all evaluated patients, 35.3% required prolonged follow-up, with an average of five visits per patient, 31 specialty referrals, and high-cost complementary tests such as CT, MRI, or transthoracic echocardiography. These costs were lower than those reported in other studies, mainly because many patients became asymptomatic within a short period of time, limiting direct comparability of results.

This healthcare burden highlights the need to establish follow-up protocols stratified by disease severity and clinical profile to optimize resource utilization and avoid unnecessary testing [51]. Furthermore, prolonged follow-up allowed the detection of alternative diagnoses, such as lung cancer or exacerbations of chronic diseases, underscoring the importance of maintaining diagnostic vigilance and avoiding misattribution of symptoms.

This study has several limitations that should be acknowledged. First, its retrospective design and single-center setting, and the number of symptomatic patients limit the generalizability of the findings and could have led to an underestimation of the actual burden of persistent symptoms and risk factors. Moreover, admission criteria during the first pandemic wave were not standardized and evolved over time, which may have biased the sample toward less severe cases.

Second, symptom collection was performed through telephone interviews using open-ended questions rather than validated questionnaires. This approach may have underestimated the prevalence of residual manifestations such as anosmia, cognitive impairment (“brain fog”), and fatigue.

Third, the absence of statistically significant associations between potential risk factors and the development of PCC should be interpreted with caution, as it may reflect limited statistical power rather than a true lack of correlation. The associations are purely descriptive, and causality cannot be inferred.

Finally, indirect costs such as work absenteeism, chronic medication use, or decreased productivity were not included in the economic analysis, and no systematic assessment of health-related quality of life was performed.

## 5. Conclusions

Among patients hospitalized for SARS-CoV-2 pneumonia during the first wave of the pandemic, 26.7% reported at least one persistent symptom at six months, while only 4.46% remained symptomatic at five years after discharge.

No clinical, laboratory, or radiological parameters were found to predict the long-term persistence of symptoms or the development of PCC.

The estimated direct healthcare cost of post-COVID-19 follow-up was EUR 381 per patient, underscoring the relevance of resource planning and the need for targeted, evidence-based follow-up strategies.

## Figures and Tables

**Table 1:** Description of clinical, radiological, and laboratory findings in patients evaluated in the post-COVID-19 clinic.

	Patients Evaluated in the Post-COVID-19 Clinic (*n* = 68)
Any persistent symptom *n* (%)	30 (44.1)
Headache *n* (%)	2 (6.7)
Dyspnea: *n* (%)	10 (33.3)
Cough *n* (%)	10 (33.3)
Concentration deficit *n* (%)	2 (6.7)
Myalgias *n* (%)	11 (36.7)
Anosmia *n* (%)	0
Fatigue *n* (%)	6 (20)
Others *n* (%) *	11 (36.7)
Physical examination *n*	47
Pulmonary auscultation	
Vesicular breath sounds preserved *n* (%)	44 (93.6)
Crackles *n* (%)	3 (6.4)
Rhonchi *n* (%)	0
Baseline oxygen saturation *n* (%)	97.5 (1.48)
Radiological assessment	
- Chest X-ray *n* (%)	62 (91%)
Complete resolution *n* (%)	53 (85%)
Persistent Infiltrates	
Decreased	9 (14.5)
Stable *n* (%)	0
Worsened *n* (%)	0
- CT *n*	4
Stable lesions *n*	1
Partial improvement *n*	2
Complete resolution *n*	1
Laboratory follow-up	
Prothrombin time % *media (SD)*	100 (46)
Fibrinogen mg/dL *media (SD)*	386 (73)
D-dimer ng/mL, *media (SD)*	384 (352)
AST (U/L) *media (SD)*	22 (10.7)
Creatinine mg/dL *media (SD)*	0.9 (0.3)
Leukocytes cells/μL *media (SD)*	5980 (1185)
Protein C-reactive protein mg/dL *media (SD)*	0.1 (0.13)
Ferritine ng/mL *media (SD)*	149 (55)

Note: SD = standard deviation; AST = aspartate aminotransferase. CT = computed tomography. Values are expressed as means and standard deviations (SD) or as absolute frequency and percentage (%). The chi-square test was used for categorical variables, and Student’s *t*-test or Mann–Whitney U test was applied for continuous variables. * Other symptoms include nonspecific complaints such as chest pain, dizziness, or sleep disturbances.

**Table 2:** Factors associated with the presence of symptoms at 180 days after hospital discharge.

Variables	Symptoms at Post-COVID-19 Clinic Visit		
	Yes (*n* = 30)	No (*n* = 82)	*p*
Male sex *n* (%)	17 (56.7%)	48 (58.5%)	0.859
Mean age, years *(SD)*	56.97 (14.6)	58.6 (16.55)	0.597
Hypertension *n* (%)	8 (26.7%)	29 (35.4%)	0.386
Diabetes mellitus *n* (%)	3 (10%)	12 (14.6%)	0.54
Dyslipidemia *n* (%)	5 (16.7%)	25 (30.5%)	0.144
Hospital admission			
Oxygen requirements *n* (%)			
None	20 (69%)	52 (65%)	0.794
Nasal cannula/Venturi mask	9 (31%)	27 (33.8%)	
Reservoir mask	0	1 (1.3%)	
Length of stay, days mean *(SD)*	6.8 (3.3)	8.9 (12.4)	0.464
Lopinavir/ritonavir *n* (%)	10 (33.3%)	34 (41.5%)	0.435
Hydroxychloroquine *n* (%)	27 (90%)	63 (76.8%)	0.12
Interferon Beta-1B *n* (%)	16 (53.3%)	47 (57.3%)	0.707
Tocilizumab *n* (%)	1 (3.3%)	1 (1.2%)	0.454
Systemic corticosteroids *n* (%)	3 (10%)	8 (9.8%)	0.969
Azithromycin *n* (%)	19 (63.3%)	38 (48.7%)	0.173
Anticoagulation *n* (%)	10 (33.3%)	27 (34.2%)	0.934
Laboratory parameters during admission			
INR (ratio) *mean (SD)*	1.38 (1.01)	0.13 (1.17)	0.7
Fibrinogen (mg/dL) *mean (SD)*	718.8 (135.2)	138 (676.83)	0.28
D-dimer (ng/mL) *mean (SD)*	1027 (1388.71)	903 (1083.59)	0.24
Creatinine (mg/dL) *mean (SD)*	0.93 (0.69)	0.4 (1)	0.03
LDH (U/L) *mean (SD)*	316.5 (114.5)	83.5 (316.31)	0.96
CRP (mg/dL) *mean (SD)*	7.77 (6.36)	6.31 (7.41)	0.86
Procalcitonin (ng/mL) *mean (SD)*	0.09 (0.04)	2.45 (0.51)	0.76
Lymphocytes (cells/μL) *mean (SD)*	1.38 (0.57)	0.55 (1.44)	0.72

Note: INR: international normalized ratio; CRP: C-reactive protein; PCT: procalcitonin; LDH: lactate dehydrogenase. Values are expressed as means and standard deviations (SD) or as absolute counts and percentages (%). The chi-square test was used for categorical variables, and Student’s *t*-test or the Mann–Whitney U test for continuous variables.

**Table 3:** Five-year follow-up characteristics of symptomatic patients.

	Patient 1	Patient 2	Patient 3
Sex	Male	Female	Male
Age *(years)*	47	64	73
Past medical history	No	No	Chronic ischemic heart disease
Hospital admission			
Oxygen requirements in the Emergency Department	No	Yes Nasal cannula	No
Length of hospital stay (*days)*	16	9	11
In-hospital complications	No	No	No
Treatment received:			
- Hydroxychloroquine	Yes	Yes	Yes
- Lopinavir/ritonavir	Yes	No	No
- Interferon Beta-1B	Yes	Yes	Yes
- Tocilizumab	No	No	No
- Systemic corticosteroids	No	No	No
- Anticoagulation	No	Yes	Yes
In-person POST-COVID-19 follow-up at 6 months			
Symptoms	Dyspnea, fatigue, and myalgia	Dyspnea, lack of concentration, and myalgia	Dyspnea and arthralgia
Pulmonary auscultation	Crackles	Normal	Crackles
Imaging findings	Normalized	Normalized	Persistent infiltrates Reduced
Analytical parameters	Normalized	Normalized	Normalized
Five-year follow-up			
Symptoms	Fatigue, vision loss	Anosmia, ageusia, fatigue	Dyspnea, arthralgia
Number of POST-COVID-19 visits	14	6	9
Number of referrals	6	4	0
Number of complementary tests	20	14	13

Note: This table summarizes the individual clinical profiles, analytical, and imaging findings of patients who continued to experience symptoms five years after hospital discharge.

**Table 4:** Distribution of healthcare costs associated with post-COVID-19 follow-up (*n* = 68).

	*N*	Unit Cost (EUR)	Total Cost (EUR)
Post-COVID-19 clinic assessments *n*	177	84.98	11,633.03
Referrals to other specialties *n*	31	84.98	2634.38
Cardiology	3		
Vascular surgery	2		
Nephrology	2		
Otolaryngology	1		
Neurology	5		
Gastroenterology	1		
Rehabilitation	2		
Ophthalmology	1		
Endocrinology	1		
Hematology	5		
Psychiatry	2		
Rheumatology	1		
Gynecology	1		
Oncology	1		
Urology	1		
General surgery	1		
Complementary diagnostic tests			7360.09
Chest X-ray	12	26.64	319.68
Chest CT scan	12	121.05	1452.6
CT scan (other sites)	1	180.66	180.66
Ultrasound	6	49.81	298.86
Brain MRI	5	232.77	1163.85
Transthoracic echocardiogram	9	140.7	1266.3
Pulmonary function tests	24	75.94	1822.56
Six-minute walk test	4	116.62	466.48
Neurophysiology	2	194.55	389.1
Cumulative total			21,627.50
Cost per patient			318

Note: Breakdown of healthcare expenditure generated by the 68 patients with in-person follow-up at the specific post-COVID-19 clinic of the Central Defense Hospital. Unit prices were obtained from the official tariff database of the Madrid Health Service (SERMAS, 2023).

## Data Availability

The data presented in this study are available on reasonable request from the corresponding author. The data are not publicly available due to privacy and ethical restrictions.

## Supplementary Materials

The following supporting information can be downloaded at https://www.mdpi.com/article/10.3390/idr18010008/s1; Supplementary File S1: telephone consultation list.

## Abbreviations

The following abbreviations are used in this manuscript:

PCC	Post-COVID-19 condition
AAPM&R	American Academy of Physical Medicine and Rehabilitation
AION	Anterior ischemic optic neuropathy
AST	Aspartate aminotransferase
COVID-19	Coronavirus disease 2019
CRP	C-reactive protein
CT	Computed tomography
HCIS	Hospital Clinical Information System
IQR	Interquartile range
LDH	Lactate dehydrogenase
MD	Median
MRI	Magnetic resonance imaging
PCR	Polymerase chain reaction
PCT	Procalcitonin
SARS-CoV-2	Severe acute respiratory syndrome coronavirus 2
SD	Standard deviation
SPSS	Statistical Package for the Social Sciences
WHO	World Health Organization
