# Supplementary material for: Persistence of Symptoms and Long-Term Recovery in Hospitalized COVID-19 Patients: Results from a Five-Year Follow-Up Cohort
Source: Infect Dis Rep. 2026 Jan 9;18(1):8. doi: 10.3390/idr18010008 (PMC12821513; doi:10.3390/idr18010008)
Supplement: Supplementary file 1 [file idr-18-00008-s001.zip › idr-4017266-supplementary.pdf]

Telephone Consultation Checklist

Item	Yes	No
**REASON FOR CONSULTATION**		
Fever		
Cough		
Dyspnea		
Chest pain		
Headache		
Sore throat		
Ageusia (loss of taste)		
Anosmia (loss of smell)		
Nausea/vomiting		
Diarrhea		
Dermatological alterations		
Neurological deterioration / Stroke		
Others		
**CLINICAL ASSESSMENT**		
Confirmed COVID-19		
Probable COVID-19		
Respiratory failure		
Pneumonia		
Thromboembolic disease / distal thrombotic events		
Heart failure		

Ischemic heart disease		
Myocarditis		
Pericarditis		
Confusional syndrome		
Stroke		
Renal failure at discharge		
Peripheral polyneuropathy		
Myopathy		
ICU admission		
**LABORATORY FINDINGS**		
LDH > 300 U/L		
Ferritin > 1000 ng/mL		
D-dimer > 1000 ng/mL		
Lymphopenia < 900 cells/μL		
AST > twice the upper limit of normal		
Troponin > 50 ng/L		
Pro-BNP > 800 or >25% increase from baseline		
Serum sodium < 135 mEq/L		
Serum sodium > 145 mEq/L		
Serum potassium < 3.5 mEq/L		
Serum potassium > 5.5 mEq/L		
**CHEST IMAGING AT ADMISSION**		

Interstitial infiltrates		
Consolidations		
Pleural effusion		
Pulmonary thromboembolism		
Others		
**TREATMENT**		
Hydroxychloroquine		
Azithromycin		
Lopinavir/ritonavir		
Corticosteroids		
Tocilizumab		
Baricitinib		
Anakinra		
Cyclosporine		
CPAP		
Interferon		
Prophylactic LMWH		
Therapeutic-dose LMWH		
Initiation of anticoagulation after admission		

Abbreviations: LDH – lactate dehydrogenase; AST – aspartate aminotransferase; CRP – C-reactive protein; BNP – B-type natriuretic peptide; Pro-BNP – N-terminal pro–B-type natriuretic peptide; LMWH – low-molecular-weight heparin; CPAP – continuous positive airway pressure; ICU – intensive care unit; U/L – units per liter; ng/mL – nanograms per milliliter; ng/L – nanograms per liter; mEq/L – milliequivalents per liter; μL – microliter.
